# 2,5-Bis(9*H*-carbazol-9-yl)thio­phene

**DOI:** 10.1107/S1600536808039767

**Published:** 2008-11-29

**Authors:** Xu-Liang Jiang, Er-Fang Huang, Guo-Wu Rao

**Affiliations:** a School of Pharmaceutical Engineering, Shenyang Pharmaceutical University, Shenyang, 110016, People’s Republic of China; b College of Pharmaceutical Science, Zhejiang University of Technology, Hangzhou, 310032, People’s Republic of China

## Abstract

The mol­ecules of the title compound, C_28_H_18_N_2_S, are built up from two triply-fused rings and one five-membered ring, with dihedral angles of 66.12 (8) and 70.96 (7)° between the central thio­phene ring and the two triply-fused rings.

## Related literature

For dicarbazolyl derivatives as potential blue-emitting hole-transporting materials, see: Wu *et al.* (2000 ▶, 2001 ▶).
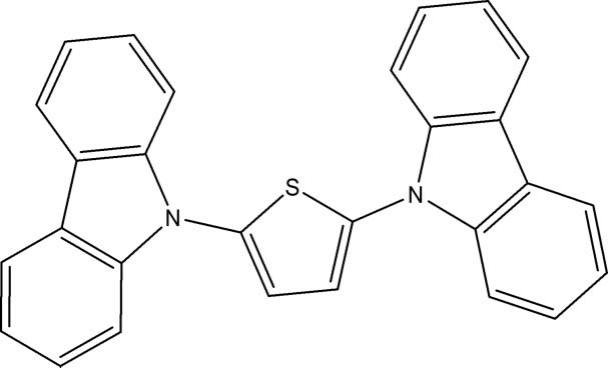


## Experimental

### 

#### Crystal data

C_28_H_18_N_2_S
*M* *_r_* = 414.50
Orthorhombic, 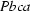

*a* = 7.8760 (16) Å
*b* = 16.098 (3) Å
*c* = 33.986 (7) Å
*V* = 4309.1 (15) Å^3^
*Z* = 8
Mo *K*α radiation
μ = 0.17 mm^−1^
*T* = 298 (2) K
0.20 × 0.18 × 0.16 mm

#### Data collection

Rigaku R-AXIS-IV diffractometer
Absorption correction: multi-scan (*ABSCOR*; Higashi, 1995 ▶) *T*_min_ = 0.963, *T*_max_ = 0.977
12051 measured reflections
3931 independent reflections
3230 reflections with *I* > 2σ(*I*)
*R*_int_ = 0.091
3 standard reflections frequency: 60 min intensity decay: 0.3%

#### Refinement

*R*[*F* ^2^ > 2σ(*F* ^2^)] = 0.063
*wR*(*F* ^2^) = 0.152
*S* = 1.13
3931 reflections
281 parameters
H-atom parameters constrained
Δρ_max_ = 0.22 e Å^−3^
Δρ_min_ = −0.23 e Å^−3^

### 

Data collection: *R-AXIS* (Rigaku, 1996 ▶); cell refinement: *R-AXIS*; data reduction: *R-AXIS* program(s) used to solve structure: *SHELXS97* (Sheldrick, 2008 ▶); program(s) used to refine structure: *SHELXL97* (Sheldrick, 2008 ▶); molecular graphics: *ORTEP-3 for Windows* (Farrugia, 1997 ▶); software used to prepare material for publication: *WinGX* (Farrugia, 1999 ▶).

## Supplementary Material

Crystal structure: contains datablocks I, global. DOI: 10.1107/S1600536808039767/dn2409sup1.cif

Structure factors: contains datablocks I. DOI: 10.1107/S1600536808039767/dn2409Isup2.hkl

Additional supplementary materials:  crystallographic information; 3D view; checkCIF report

## Figures and Tables

**Fig. 1.**
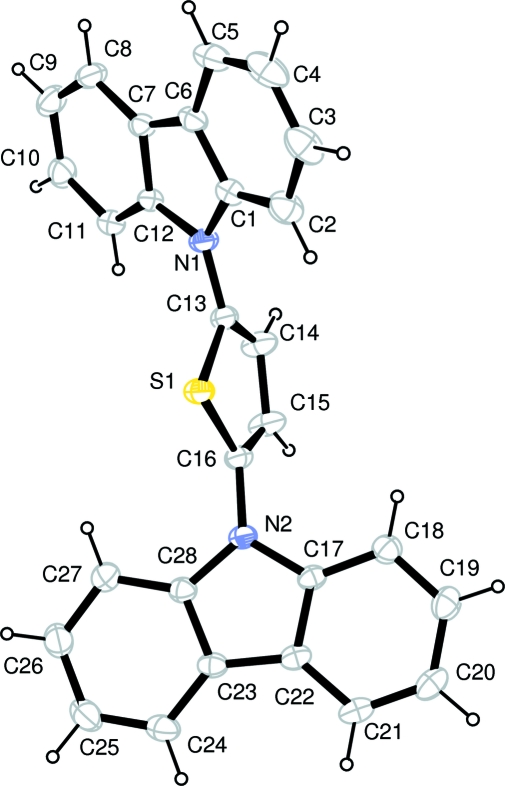
The structure of (I), showing the atom-labelling scheme. Ellipsoids are drawn at the 30% probability level.

## supplementary crystallographic information

### Comment

Due to the great potential in flat-panel displays, organic light-emitting diodes (OLEDs) have been received continuous attention for years. Dicarbazolyl derivatives bridged by various aromatic spacers could emit blue light in solution, and could be used as excellent blue-emitting hole-transporting materials (Wu *et al.*, 2000, 2001). As dicarbazolyl derivatives are of great importance in electroluminescent devices, we have undertaken the crystal structure determination of the title compound.

The molecular (I) is built up from two three-fused rings and one five-membered ring. (Fig. 1). The three fused rings are coplanar within 0.0306 (27) and 0.0288 (26) Å, respectively. The five-membered ring is coplanar within 0.0027 (18) Å. The dihedral angles between the thiophene ring and the three-fused rings are 66.12 (8) and 70.96 (7)°, respectively.

### Experimental

2,5-dibromothiophene (4.84 g, 20.0 mmol), 9*H*-carbazole (6.69 g, 40.0 mmol), sodium *tert*-butoxide (4.61 g, 48.0 mmol), Pd(OAc)_2_ (90 mg, 0.4 mmol), P(t—Bu)_3_ (0.4 ml, 1.6 mmol, 0.81 *M* in *o*-xylene) and dry *o*-xylene (60 ml) were placed in a round-bottomed flask, and the solution was stirred at reflux for 72 h. After cooling, 2 ml of water was added, and the solution was then pumped dry, and the residue was extracted with dichloromethane/water, filtered and dried over magnesium sulfate. The pure product (m. p. 250–252°C) was obtained through silica gel chromatography (eluant: petroleum ether). A solution of the compound in n-hexane/dichloromethane (*v*/*v*=5/1) was concentrated gradually at room temperature to afford colorless prisms.

### Refinement

H atoms were included in calculated positions and refined using a riding model. H atoms were given isotropic displacement parameters equal to 1.2 times the equivalent isotropic displacement parameters of their parent atoms and C—H distances were restrained to 0.93 Å.

### Crystal data

**Table:**

C_28_H_18_N_2_S	*F*_000_ = 1728
*M**_r_* = 414.50	*D*_x_ = 1.278 Mg m^−^^3^
Orthorhombic, *P**b**c**a*	Mo *K*α radiation λ = 0.71073 Å
Hall symbol: -P 2ac 2ab	Cell parameters from 398 reflections
*a* = 7.8760 (16) Å	θ = 2–25.1º
*b* = 16.098 (3) Å	µ = 0.17 mm^−^^1^
*c* = 33.986 (7) Å	*T* = 298 (2) K
*V* = 4309.1 (15) Å^3^	Prismatic, colorless
*Z* = 8	0.20 × 0.18 × 0.16 mm

### Data collection

**Table:**

Rigaku R-AXIS-IV diffractometer	*R*_int_ = 0.091
Radiation source: fine-focus sealed tube	θ_max_ = 25.5º
Monochromator: graphite	θ_min_ = 1.2º
*T* = 298(2) K	*h* = −9→9
Oscillation frames scans	*k* = −19→0
Absorption correction: multi-scan(ABSCOR; Higashi, 1995)	*l* = −41→41
*T*_min_ = 0.963, *T*_max_ = 0.977	3 standard reflections
12051 measured reflections	every 60 min
3931 independent reflections	intensity decay: 0.3%
3230 reflections with *I* > 2σ(*I*)	

### Refinement

**Table:**

Refinement on *F*^2^	Hydrogen site location: inferred from neighbouring sites
Least-squares matrix: full	H-atom parameters constrained
*R*[*F*^2^ > 2σ(*F*^2^)] = 0.063	*w* = 1/[σ^2^(*F*_o_^2^) + (0.0561*P*)^2^ + 1.4309*P*] where *P* = (*F*_o_^2^ + 2*F*_c_^2^)/3
*wR*(*F*^2^) = 0.152	(Δ/σ)_max_ = 0.005
*S* = 1.13	Δρ_max_ = 0.22 e Å^−^^3^
3931 reflections	Δρ_min_ = −0.23 e Å^−^^3^
281 parameters	Extinction correction: SHELXL97 (Sheldrick, 2008), Fc^*^=kFc[1+0.001xFc^2^λ^3^/sin(2θ)]^-1/4^
Primary atom site location: structure-invariant direct methods	Extinction coefficient: 0.0075 (8)
Secondary atom site location: difference Fourier map	

### Special details

**Table:**

Geometry. All e.s.d.'s (except the e.s.d. in the dihedral angle between two l.s. planes) are estimated using the full covariance matrix. The cell e.s.d.'s are taken into account individually in the estimation of e.s.d.'s in distances, angles and torsion angles; correlations between e.s.d.'s in cell parameters are only used when they are defined by crystal symmetry. An approximate (isotropic) treatment of cell e.s.d.'s is used for estimating e.s.d.'s involving l.s. planes.
Refinement. Refinement of *F*^2^ against ALL reflections. The weighted *R*-factor *wR* and goodness of fit *S* are based on *F*^2^, conventional *R*-factors *R* are based on *F*, with *F* set to zero for negative *F*^2^. The threshold expression of *F*^2^ > σ(*F*^2^) is used only for calculating *R*-factors(gt) *etc*. and is not relevant to the choice of reflections for refinement. *R*-factors based on *F*^2^ are statistically about twice as large as those based on *F*, and *R*- factors based on ALL data will be even larger.

### Fractional atomic coordinates and isotropic or equivalent isotropic displacement parameters (Å^2^)

**Table:**

	*x*	*y*	*z*	*U*_iso_*/*U*_eq_	
S1	0.42510 (9)	0.38837 (4)	0.134571 (19)	0.0563 (2)	
N1	0.1144 (3)	0.45028 (13)	0.15532 (6)	0.0547 (5)	
N2	0.5972 (3)	0.31480 (13)	0.07385 (6)	0.0528 (5)	
C1	0.1385 (3)	0.52907 (16)	0.17188 (8)	0.0549 (6)	
C2	0.2565 (4)	0.5891 (2)	0.16243 (11)	0.0778 (9)	
H2A	0.3375	0.5804	0.1429	0.093*	
C3	0.2491 (5)	0.6626 (2)	0.18321 (12)	0.0908 (11)	
H3A	0.3271	0.7043	0.1776	0.109*	
C4	0.1297 (6)	0.6760 (2)	0.21206 (12)	0.0935 (12)	
H4A	0.1285	0.7264	0.2254	0.112*	
C5	0.0140 (5)	0.6171 (2)	0.22129 (9)	0.0756 (9)	
H5A	−0.0666	0.6270	0.2407	0.091*	
C6	0.0166 (3)	0.54065 (17)	0.20123 (7)	0.0552 (6)	
C7	−0.0844 (3)	0.46647 (17)	0.20323 (7)	0.0536 (6)	
C8	−0.2183 (4)	0.4410 (2)	0.22699 (8)	0.0706 (8)	
H8A	−0.2620	0.4764	0.2461	0.085*	
C9	−0.2852 (4)	0.3632 (2)	0.22198 (9)	0.0797 (9)	
H9A	−0.3736	0.3454	0.2381	0.096*	
C10	−0.2226 (4)	0.3107 (2)	0.19319 (9)	0.0717 (8)	
H10A	−0.2705	0.2582	0.1902	0.086*	
C11	−0.0908 (4)	0.33396 (17)	0.16874 (8)	0.0595 (7)	
H11A	−0.0508	0.2988	0.1491	0.071*	
C12	−0.0208 (3)	0.41184 (15)	0.17466 (7)	0.0484 (6)	
C13	0.2153 (3)	0.41352 (16)	0.12579 (7)	0.0518 (6)	
C14	0.1618 (4)	0.3893 (2)	0.08962 (8)	0.0721 (9)	
H14A	0.0525	0.3978	0.0801	0.087*	
C15	0.2932 (4)	0.3495 (2)	0.06807 (8)	0.0737 (9)	
H15A	0.2791	0.3287	0.0427	0.088*	
C16	0.4400 (3)	0.34488 (16)	0.08817 (7)	0.0517 (6)	
C17	0.6898 (3)	0.35122 (15)	0.04319 (7)	0.0484 (6)	
C18	0.6625 (4)	0.42622 (17)	0.02422 (8)	0.0626 (7)	
H18A	0.5728	0.4608	0.0312	0.075*	
C19	0.7746 (4)	0.44742 (19)	−0.00553 (9)	0.0710 (8)	
H19A	0.7599	0.4975	−0.0187	0.085*	
C20	0.9084 (4)	0.3958 (2)	−0.01618 (9)	0.0715 (8)	
H20A	0.9806	0.4114	−0.0365	0.086*	
C21	0.9348 (3)	0.32234 (19)	0.00301 (8)	0.0625 (7)	
H21A	1.0244	0.2879	−0.0042	0.075*	
C22	0.8259 (3)	0.29954 (16)	0.03348 (7)	0.0511 (6)	
C23	0.8173 (3)	0.22830 (16)	0.05942 (7)	0.0520 (6)	
C24	0.9152 (4)	0.1575 (2)	0.06395 (9)	0.0711 (8)	
H24A	1.0113	0.1493	0.0486	0.085*	
C25	0.8682 (5)	0.0997 (2)	0.09146 (10)	0.0838 (10)	
H25A	0.9330	0.0518	0.0945	0.101*	
C26	0.7258 (5)	0.1112 (2)	0.11483 (9)	0.0814 (9)	
H26A	0.6974	0.0710	0.1333	0.098*	
C27	0.6255 (4)	0.18089 (18)	0.11129 (8)	0.0673 (8)	
H27A	0.5297	0.1884	0.1269	0.081*	
C28	0.6737 (3)	0.23931 (16)	0.08351 (7)	0.0518 (6)	

### Atomic displacement parameters (Å^2^)

**Table:**

	*U*^11^	*U*^22^	*U*^33^	*U*^12^	*U*^13^	*U*^23^
S1	0.0515 (4)	0.0642 (4)	0.0531 (4)	0.0098 (3)	0.0019 (3)	−0.0067 (3)
N1	0.0541 (12)	0.0513 (12)	0.0586 (12)	0.0035 (10)	0.0152 (10)	−0.0071 (10)
N2	0.0474 (11)	0.0548 (12)	0.0564 (12)	0.0049 (10)	0.0114 (9)	−0.0021 (10)
C1	0.0545 (15)	0.0498 (15)	0.0604 (15)	0.0060 (12)	−0.0023 (12)	−0.0032 (12)
C2	0.068 (2)	0.0620 (19)	0.103 (2)	−0.0023 (16)	0.0013 (17)	−0.0029 (17)
C3	0.083 (3)	0.0552 (19)	0.134 (3)	−0.0055 (17)	−0.020 (2)	−0.005 (2)
C4	0.098 (3)	0.064 (2)	0.119 (3)	0.017 (2)	−0.036 (2)	−0.027 (2)
C5	0.080 (2)	0.073 (2)	0.074 (2)	0.0289 (18)	−0.0145 (16)	−0.0226 (16)
C6	0.0568 (15)	0.0560 (15)	0.0529 (14)	0.0156 (13)	−0.0088 (12)	−0.0073 (12)
C7	0.0528 (15)	0.0630 (16)	0.0451 (13)	0.0201 (13)	−0.0010 (11)	−0.0001 (11)
C8	0.0618 (18)	0.096 (2)	0.0544 (16)	0.0204 (17)	0.0139 (13)	−0.0022 (15)
C9	0.0617 (19)	0.106 (3)	0.071 (2)	0.0008 (19)	0.0196 (15)	0.0159 (19)
C10	0.0631 (18)	0.0702 (19)	0.082 (2)	−0.0038 (15)	0.0063 (15)	0.0152 (16)
C11	0.0583 (16)	0.0564 (16)	0.0638 (16)	0.0087 (13)	0.0070 (12)	−0.0001 (12)
C12	0.0459 (13)	0.0514 (14)	0.0481 (13)	0.0100 (11)	0.0048 (10)	0.0005 (11)
C13	0.0488 (14)	0.0532 (14)	0.0533 (14)	0.0030 (12)	0.0089 (11)	−0.0029 (11)
C14	0.0489 (15)	0.107 (2)	0.0605 (17)	0.0097 (16)	0.0022 (12)	−0.0179 (16)
C15	0.0494 (16)	0.112 (3)	0.0594 (17)	0.0088 (17)	0.0050 (13)	−0.0236 (17)
C16	0.0455 (14)	0.0553 (15)	0.0542 (14)	0.0021 (11)	0.0094 (11)	−0.0036 (11)
C17	0.0457 (13)	0.0510 (14)	0.0486 (13)	−0.0043 (11)	0.0042 (10)	−0.0095 (11)
C18	0.0665 (18)	0.0560 (16)	0.0652 (17)	−0.0013 (14)	0.0058 (13)	−0.0043 (13)
C19	0.077 (2)	0.0639 (18)	0.0724 (18)	−0.0182 (16)	0.0076 (16)	0.0036 (15)
C20	0.0661 (19)	0.084 (2)	0.0645 (17)	−0.0250 (17)	0.0170 (14)	−0.0064 (16)
C21	0.0470 (15)	0.0776 (19)	0.0629 (16)	−0.0076 (14)	0.0120 (12)	−0.0205 (15)
C22	0.0447 (13)	0.0586 (15)	0.0501 (13)	−0.0056 (11)	0.0025 (10)	−0.0168 (12)
C23	0.0495 (14)	0.0572 (15)	0.0494 (13)	0.0041 (12)	−0.0014 (11)	−0.0134 (12)
C24	0.0687 (19)	0.077 (2)	0.0676 (18)	0.0213 (16)	−0.0006 (14)	−0.0157 (16)
C25	0.105 (3)	0.071 (2)	0.076 (2)	0.0317 (19)	−0.0114 (19)	−0.0026 (17)
C26	0.109 (3)	0.069 (2)	0.0669 (19)	0.0126 (19)	0.0003 (19)	0.0067 (16)
C27	0.0769 (19)	0.0672 (18)	0.0579 (16)	0.0052 (15)	0.0090 (14)	0.0010 (14)
C28	0.0528 (14)	0.0536 (15)	0.0490 (14)	0.0038 (12)	0.0012 (11)	−0.0067 (11)

### Geometric parameters (Å, °)

**Table:**

S1—C13	1.727 (3)	C11—H11A	0.9300
S1—C16	1.729 (3)	C13—C14	1.356 (4)
N1—C12	1.396 (3)	C14—C15	1.421 (4)
N1—C1	1.401 (3)	C14—H14A	0.9300
N1—C13	1.410 (3)	C15—C16	1.345 (4)
N2—C28	1.396 (3)	C15—H15A	0.9300
N2—C17	1.401 (3)	C17—C18	1.385 (4)
N2—C16	1.415 (3)	C17—C22	1.396 (3)
C1—C2	1.379 (4)	C18—C19	1.385 (4)
C1—C6	1.397 (4)	C18—H18A	0.9300
C2—C3	1.379 (5)	C19—C20	1.390 (4)
C2—H2A	0.9300	C19—H19A	0.9300
C3—C4	1.376 (6)	C20—C21	1.366 (4)
C3—H3A	0.9300	C20—H20A	0.9300
C4—C5	1.352 (5)	C21—C22	1.394 (4)
C4—H4A	0.9300	C21—H21A	0.9300
C5—C6	1.407 (4)	C22—C23	1.448 (4)
C5—H5A	0.9300	C23—C24	1.385 (4)
C6—C7	1.437 (4)	C23—C28	1.408 (3)
C7—C8	1.390 (4)	C24—C25	1.370 (5)
C7—C12	1.403 (3)	C24—H24A	0.9300
C8—C9	1.370 (5)	C25—C26	1.387 (5)
C8—H8A	0.9300	C25—H25A	0.9300
C9—C10	1.384 (4)	C26—C27	1.377 (4)
C9—H9A	0.9300	C26—H26A	0.9300
C10—C11	1.381 (4)	C27—C28	1.386 (4)
C10—H10A	0.9300	C27—H27A	0.9300
C11—C12	1.384 (4)		
			
C13—S1—C16	90.13 (12)	C13—C14—C15	111.8 (3)
C12—N1—C1	108.4 (2)	C13—C14—H14A	124.1
C12—N1—C13	125.4 (2)	C15—C14—H14A	124.1
C1—N1—C13	126.1 (2)	C16—C15—C14	112.9 (3)
C28—N2—C17	108.34 (19)	C16—C15—H15A	123.6
C28—N2—C16	126.5 (2)	C14—C15—H15A	123.6
C17—N2—C16	124.6 (2)	C15—C16—N2	126.6 (2)
C2—C1—C6	122.4 (3)	C15—C16—S1	112.49 (19)
C2—C1—N1	129.2 (3)	N2—C16—S1	120.76 (18)
C6—C1—N1	108.3 (2)	C18—C17—C22	121.9 (2)
C3—C2—C1	117.0 (3)	C18—C17—N2	129.1 (2)
C3—C2—H2A	121.5	C22—C17—N2	109.0 (2)
C1—C2—H2A	121.5	C19—C18—C17	117.1 (3)
C4—C3—C2	121.9 (4)	C19—C18—H18A	121.5
C4—C3—H3A	119.0	C17—C18—H18A	121.5
C2—C3—H3A	119.0	C18—C19—C20	121.7 (3)
C5—C4—C3	121.1 (3)	C18—C19—H19A	119.1
C5—C4—H4A	119.5	C20—C19—H19A	119.1
C3—C4—H4A	119.5	C21—C20—C19	120.5 (3)
C4—C5—C6	119.4 (3)	C21—C20—H20A	119.7
C4—C5—H5A	120.3	C19—C20—H20A	119.7
C6—C5—H5A	120.3	C20—C21—C22	119.3 (3)
C1—C6—C5	118.2 (3)	C20—C21—H21A	120.4
C1—C6—C7	107.7 (2)	C22—C21—H21A	120.4
C5—C6—C7	134.1 (3)	C21—C22—C17	119.4 (3)
C8—C7—C12	119.2 (3)	C21—C22—C23	133.6 (2)
C8—C7—C6	133.8 (3)	C17—C22—C23	107.0 (2)
C12—C7—C6	106.9 (2)	C24—C23—C28	119.1 (3)
C9—C8—C7	119.3 (3)	C24—C23—C22	133.9 (2)
C9—C8—H8A	120.4	C28—C23—C22	107.0 (2)
C7—C8—H8A	120.4	C25—C24—C23	119.0 (3)
C8—C9—C10	120.6 (3)	C25—C24—H24A	120.5
C8—C9—H9A	119.7	C23—C24—H24A	120.5
C10—C9—H9A	119.7	C24—C25—C26	121.2 (3)
C11—C10—C9	121.8 (3)	C24—C25—H25A	119.4
C11—C10—H10A	119.1	C26—C25—H25A	119.4
C9—C10—H10A	119.1	C27—C26—C25	121.5 (3)
C10—C11—C12	117.2 (3)	C27—C26—H26A	119.2
C10—C11—H11A	121.4	C25—C26—H26A	119.2
C12—C11—H11A	121.4	C26—C27—C28	117.1 (3)
C11—C12—N1	129.6 (2)	C26—C27—H27A	121.5
C11—C12—C7	121.7 (2)	C28—C27—H27A	121.5
N1—C12—C7	108.7 (2)	C27—C28—N2	129.3 (2)
C14—C13—N1	126.2 (2)	C27—C28—C23	122.1 (2)
C14—C13—S1	112.72 (19)	N2—C28—C23	108.6 (2)
N1—C13—S1	120.95 (19)		
			
C12—N1—C1—C2	179.9 (3)	C14—C15—C16—N2	−174.8 (3)
C13—N1—C1—C2	3.4 (5)	C14—C15—C16—S1	0.4 (4)
C12—N1—C1—C6	−0.9 (3)	C28—N2—C16—C15	−106.6 (4)
C13—N1—C1—C6	−177.4 (2)	C17—N2—C16—C15	63.8 (4)
C6—C1—C2—C3	−0.4 (5)	C28—N2—C16—S1	78.6 (3)
N1—C1—C2—C3	178.6 (3)	C17—N2—C16—S1	−111.0 (2)
C1—C2—C3—C4	−0.1 (5)	C13—S1—C16—C15	−0.4 (3)
C2—C3—C4—C5	0.1 (6)	C13—S1—C16—N2	175.1 (2)
C3—C4—C5—C6	0.4 (5)	C28—N2—C17—C18	−179.5 (3)
C2—C1—C6—C5	0.9 (4)	C16—N2—C17—C18	8.6 (4)
N1—C1—C6—C5	−178.3 (2)	C28—N2—C17—C22	−0.5 (3)
C2—C1—C6—C7	180.0 (3)	C16—N2—C17—C22	−172.3 (2)
N1—C1—C6—C7	0.8 (3)	C22—C17—C18—C19	1.2 (4)
C4—C5—C6—C1	−0.9 (4)	N2—C17—C18—C19	−179.9 (3)
C4—C5—C6—C7	−179.6 (3)	C17—C18—C19—C20	0.3 (4)
C1—C6—C7—C8	177.8 (3)	C18—C19—C20—C21	−0.9 (5)
C5—C6—C7—C8	−3.4 (5)	C19—C20—C21—C22	0.0 (4)
C1—C6—C7—C12	−0.3 (3)	C20—C21—C22—C17	1.4 (4)
C5—C6—C7—C12	178.6 (3)	C20—C21—C22—C23	−179.8 (3)
C12—C7—C8—C9	0.0 (4)	C18—C17—C22—C21	−2.1 (4)
C6—C7—C8—C9	−177.9 (3)	N2—C17—C22—C21	178.8 (2)
C7—C8—C9—C10	−1.0 (5)	C18—C17—C22—C23	178.8 (2)
C8—C9—C10—C11	0.4 (5)	N2—C17—C22—C23	−0.3 (3)
C9—C10—C11—C12	1.3 (4)	C21—C22—C23—C24	1.2 (5)
C10—C11—C12—N1	178.2 (3)	C17—C22—C23—C24	−179.9 (3)
C10—C11—C12—C7	−2.4 (4)	C21—C22—C23—C28	−178.0 (3)
C1—N1—C12—C11	−179.8 (3)	C17—C22—C23—C28	0.9 (3)
C13—N1—C12—C11	−3.2 (4)	C28—C23—C24—C25	0.8 (4)
C1—N1—C12—C7	0.7 (3)	C22—C23—C24—C25	−178.3 (3)
C13—N1—C12—C7	177.2 (2)	C23—C24—C25—C26	−0.4 (5)
C8—C7—C12—C11	1.8 (4)	C24—C25—C26—C27	0.3 (6)
C6—C7—C12—C11	−179.8 (2)	C25—C26—C27—C28	−0.4 (5)
C8—C7—C12—N1	−178.7 (2)	C26—C27—C28—N2	−179.7 (3)
C6—C7—C12—N1	−0.3 (3)	C26—C27—C28—C23	0.8 (4)
C12—N1—C13—C14	64.9 (4)	C17—N2—C28—C27	−178.5 (3)
C1—N1—C13—C14	−119.2 (3)	C16—N2—C28—C27	−6.8 (4)
C12—N1—C13—S1	−110.7 (3)	C17—N2—C28—C23	1.1 (3)
C1—N1—C13—S1	65.2 (3)	C16—N2—C28—C23	172.7 (2)
C16—S1—C13—C14	0.3 (2)	C24—C23—C28—C27	−0.9 (4)
C16—S1—C13—N1	176.5 (2)	C22—C23—C28—C27	178.4 (2)
N1—C13—C14—C15	−176.0 (3)	C24—C23—C28—N2	179.4 (2)
S1—C13—C14—C15	−0.1 (4)	C22—C23—C28—N2	−1.2 (3)
C13—C14—C15—C16	−0.2 (4)		
